# LATE-LIFE CAREGIVING IN MEXICAN AMERICAN FAMILIES

**DOI:** 10.1093/geroni/igz038.670

**Published:** 2019-11-08

**Authors:** Jaqueline L Angel

**Affiliations:** The University of Texas at Austin, Austin, Texas, United States

## Abstract

This study explores how sociological triangulation can be used to examine Mexican-American families in late-life caregiving. We examine the importance of household structure in providing dementia care. The movement away from traditional residential arrangements that result from neoliberal market reforms and international migration means that intergenerational relationship norms and exchanges will inevitably change and affect Mexican-American families caring for their elders. For many aging Mexican-Americans, a severe lack of resources and health limitations introduce major uncertainties about their futures. We employ the H-EPESE and Sacramento Area Longitudinal Study of Aging to document the nature, extent, and quality of dementia caregiving in the Southwestern United States. Qualitative results uncover how the changing meaning of social relationships impacts family life for older parents with dementia. Multivariate analyses reveal that the late-life migration (after 50yrs) undermines resources and opportunities for community-based care. Implications of the findings for informal and formal support are discussed.

